# Radial Wall Strain for Predicting Severe Dissection After Drug-Coated Balloon

**DOI:** 10.1016/j.jacasi.2026.05.035

**Published:** 2026-07-18

**Authors:** Wence Shi, Qifeng Zhu, Zhenyan Zhao, Chengqing Jiang, Zhengrong Liu, Guannan Niu, Erli Zhang, Junbo Ge, Yongjian Wu, Hongliang Zhang

**Affiliations:** a Department of Cardiology, Fuwai Hospital, National Center for Cardiovascular Disease, Chinese Academy of Medical Science and Peking Union Medical College, Beijing, China; b Department of Cardiology, The Second Affiliated Hospital, School of Medicine, Zhejiang University, Hangzhou, China

**Keywords:** coronary dissection, drug-coated balloon, percutaneous coronary intervention, radial wall strain

## Abstract

**Background:**

Although drug-coated balloons (DCBs) demonstrate efficacy in de novo coronary artery disease, patients with non–small vessel lesions (NSVLs, ≥2.75 mm) face elevated risks of rescue stenting due to procedure-related coronary dissection. Radial wall stress maximum (RWS_max_), an angiography-derived parameter reflecting plaque vulnerability, may predict dissection severity and optimize patient selection for DCB strategies.

**Objectives:**

This study aimed to evaluate the predictive value of RWS_max_ for severe coronary dissection following DCB treatment in de novo NSVL.

**Methods:**

This study retrospectively analyzed 194 patients with de novo NSVL who were evaluated for DCB angioplasty. Dissections were classified via National Heart, Lung, and Blood Institute criteria: nonsevere (no dissection or Type A/B) vs severe (Type C-F). RWS_max_ was quantified using electrocardiography-gated angiographic analysis (AngioPlus Core software). Multivariable regression and receiver operating characteristic analyses identified predictors of severe dissection.

**Results:**

Severe dissections occurred in 32.0% (62 of 194), predominantly Type C (83.9%, 52 of 62). RWS_max_ increased progressively with dissection severity (Jonckheere-Terpstra *P* < 0.001) and was higher compared with patients with nonsevere dissections (14.70% [12.80-16.65] vs 12.20% [11.00-13.30], *P* < 0.001). RWS_max_ independently predicted severe dissection (adjusted OR per 1%: 1.55; 95% CI: 1.31-1.90; *P* < 0.001), with an optimal cutoff of 13.7% (area under the curve = 0.752 [0.673-0.831], sensitivity = 61.3% [56.5% to 66.1%], specificity = 81.1%[74.4% to 87.8%]). Incorporating RWS_max_ into clinical prediction models significantly improved discrimination (Δarea under the curve = +0.093 [0.035-0.151]; *P* = 0.008). Subgroup analyses confirmed consistency across lesion types, calcification severity, and operator experience levels.

**Conclusions:**

RWS_max_ is a robust, angiography-based predictor of severe coronary dissection after DCB in NSVL. A 13.7% cutoff helps identify high-risk patients, supporting precision selection for DCB strategy.

Percutaneous coronary intervention (PCI) for coronary artery disease has undergone significant evolution, transitioning from plain balloon angioplasty to bare-metal stents and subsequently to drug-eluting stents, driven by advancements in interventional devices and antiproliferative pharmacotherapy.^1^ These developments have markedly improved the quality of life and long-term prognosis for patients with coronary artery disease.^2^^,^^3^ However, the permanent presence of these stents necessitates prolonged dual antiplatelet therapy, increasing the bleeding risk and complicating management for patients requiring time-sensitive surgical interventions.^4^ Furthermore, the persistent metallic foreign body induces chronic local inflammation and elevates the incidence of long-term in-stent restenosis.^5^ Particularly in younger patients, the technical challenges of future reinterventions become substantially amplified. These limitations have prompted serious consideration of drug-coated balloon (DCB) angioplasty as a potentially preferred interventional strategy.^6^

Emerging evidence from numerous clinical investigations supports the safety and feasibility of DCB application in de novo coronary lesions.^7^ However, DCB treatment in non–small vessel lesions (NSVL, ≥2.75 mm) is associated with an increased incidence of rescue stenting due to procedure-related coronary dissection, potentially compromising immediate therapeutic outcomes.^8^ There is an urgent clinical need to develop practical and effective preoperative screening methods to identify patients at high risk of coronary dissection, thereby avoiding DCB therapy in this vulnerable subgroup with de novo NSVL.

Radial wall strain (RWS) is an emerging angiographic index that captures the cyclic deformation of the vessel wall during the cardiac cycle by quantifying beat-to-beat changes in luminal diameter on standard 2-dimensional angiography. Its reproducibility and ease of measurement make it attractive for widespread clinical use, and automated edge-detection algorithms facilitate rapid acquisition in less than 2 minutes per lesion. Intravascular imaging studies have demonstrated that higher RWS correlates strongly with lipid-rich necrotic core volume thin-cap fibroatheroma identified on optical coherence tomography (OCT), supporting its role as a surrogate for plaque vulnerability.^9^ Previous investigations have demonstrated associations between peri-stent coronary dissection and specific plaque characteristics, including fibrous cap thickness^10^ and lipid-rich plaque.^11^ Based on these pathophysiological insights, we hypothesize that maximum radial wall stress (RWS_max_)—as a readily obtainable angiographic parameter—may predict the severity of immediate coronary dissection following DCB angioplasty. This investigation aimed to provide evidence-based support for optimizing patient selection in DCB therapy.

## Methods

### Study design and participants

This was a retrospective, single-center observational study conducted at Fuwai Hospital, aiming to identify clinical and angiographic predictors of immediate coronary dissection following DCB angioplasty in de novo NSVL. The study follows the STARD (Standards for Reporting of Diagnostic Accuracy Studies) guidelines for reporting observational clinical studies (Supplemental Figure 1). Between October 2023 and December 2024, we reviewed patients who underwent DCB angioplasty for de novo noncomplex coronary artery disease involving NSVL (reference vessel diameter ≥2.75 mm). A total of 200 consecutive patients meeting the following eligibility criteria were included in the analysis: 1) age ≥18 years; 2) presence of a single functionally significant de novo coronary lesion (≥70% diameter stenosis by visual estimation); 3) lesion suitable for balloon-based revascularization without the need for planned stent implantation; 4) nontarget lesions considered clinically insignificant, with no scheduled revascularization within 1 year as per current guidelines and operator judgment; and 5) availability of complete angiographic and procedural data. Patients were excluded if they had 1) acute coronary syndrome within the preceding 6 months; 2) prior coronary interventions (PCI, stenting, balloon angioplasty, or DCB) within 12 months; 3) established DCB indications such as in-stent restenosis or bifurcation lesions requiring side branch treatment; 4) complex anatomical features including lesion length ≥40 mm, severe calcification, significant vessel tortuosity (≥90°), chronic total occlusion, or involvement of left main or bypass grafts; or 5) significant comorbid conditions including decompensated heart failure, severe hepatic or renal impairment, active malignancy, or an estimated life expectancy of less than 1 year. The study was approved by the Institutional Review Board of Fuwai Hospital (Approval No. 2026-3072) and adhered to the principles outlined in the Declaration of Helsinki. Written informed consent was obtained from all patients for the use of their anonymized data in this analysis.

### Procedures

For patients deemed suitable for DCB treatment by the operator and meeting the inclusion criteria, the procedure was performed according to the following standards: For noncomplex lesions, it is recommended to use a semicompliant or noncompliant balloon that matches the artery diameter for lesion preparation. The balloon-to-vessel diameter ratio should be maintained at 0.8 to 1.0, with a moderate inflation pressure (8-14 atm, 1 atm = 101.325 kPa) to minimize the risk of dissection. In cases of inadequate vessel expansion, potential undersizing, or difficulty in balloon delivery, initial dilation with a smaller-diameter balloon is advised, followed by reassessment of vessel size after the administration of vasodilators. If standard semicompliant balloon dilation fails, high-pressure noncompliant balloons or scoring/cutting balloons are recommended for lesion preparation. For calcified lesions, these specialized balloons may provide more predictable and uniform predilation outcomes compared with standard balloons. Following predilation, patients receive DCB treatment, with rescue drug-eluting stent implantation if necessary.

### Assessment of target lesion characteristics

All target lesion morphological features were independently evaluated by 2 experienced interventional cardiologists using preprocedural angiography. In cases of disagreement, a third senior interventionalist provided adjudication. All assessors were blinded to postprocedural angiographic outcomes.

The TIMI (Thrombolysis In Myocardial Infarction) flow grade^12^ is used to assess the blood flow in coronary lesions, where TIMI 0 indicates a complete absence of flow, TIMI 1 signifies only minimal flow reaching the distal segment, TIMI 2 represents partial reperfusion with delayed flow, and TIMI 3 denotes a full restoration of normal flow.

Coronary lesion angulation is defined as follows: lesions with an angle of less than 45° are considered nonangulated, those with angles between 45° and 90° are classified as severe angulated lesions, and lesions with angles greater than 90° are deemed extremely angulated and are excluded from the study.

Lesions that exhibit clear high-density shadows during any phase of the cardiac cycle are considered severely calcified; lesions with significant circumferential calcification that are deemed unsuitable for effective predilation by the operator are excluded from this study.

In this study, the lesion classification is based on the American College of Cardiology/American Heart Association coronary lesion classification,^13^^,^^14^ which divides lesions into 3 types. Type A lesions are focal (<10 mm), concentric, nonangulated (<45°), with little or no calcification, nonocclusive, nonostial, without major side branch involvement, and lacking thrombus. Type B lesions are tubular (10-20 mm), exhibit moderate proximal vessel tortuosity, eccentricity, angulated lesions (45-90°), moderate to severe calcification, ostial involvement, bifurcation lesions requiring dual guide wires, and some thrombus—with Type B1 having 1 of these features and Type B2 having 2 or more. Type C lesions are defined as diffuse, with severely tortuous proximal vessels, lesion angulation >90°, difficulty in protecting major side branches, degenerated venous grafts, and fragile characteristics.

**Figure 1:**
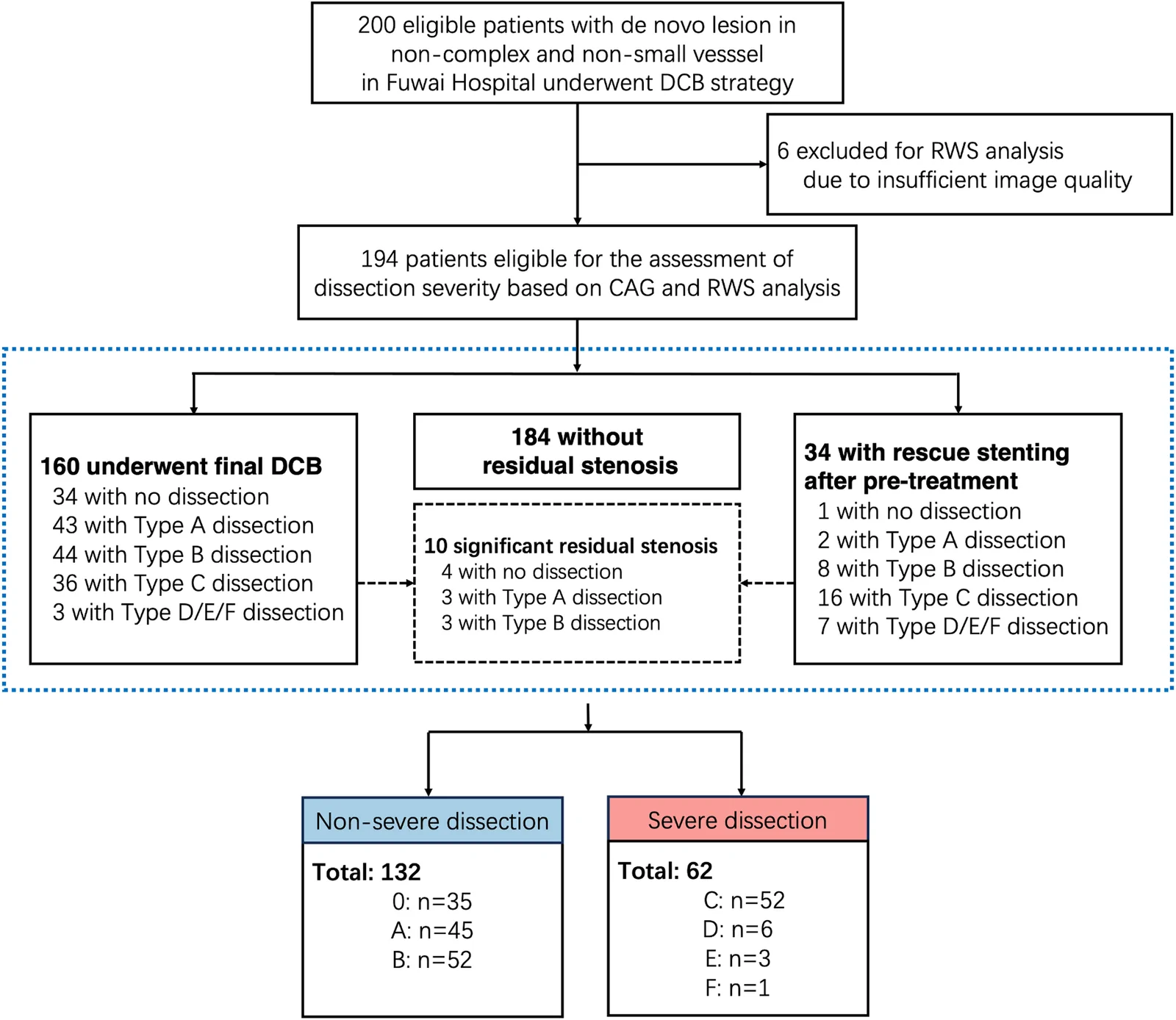
Study Profile After applying the inclusion/exclusion criteria and technical standards, a total of 194 patients were included in the analysis. The blue dashed box represents the patient grouping after standardized pretreatment. Among them, 160 patients underwent DCB treatment following pretreatment, whereas in 34 patients, the operators decided to proceed directly with rescue stenting based on intraoperative assessment. In addition, 10 patients in these 2 groups were identified as having severe residual stenosis (≥30%) on evaluation and were excluded, leaving 184 patients who achieved the expected dilation outcome. Sensitivity analyses were conducted for these 3 patient groups. Finally, the patients were further stratified based on the presence of severe dissection (Type C to D) for the final analysis. CAG = coronary angiography; DCB = drug-coated balloon; RWS = radial wall strain.

In this study, the evaluation of coronary lesion dissection using the National Heart, Lung, and Blood Institute classification is performed as follows: if the target lesion is treated with DCB after pretreatment, the degree of dissection is assessed based on the post-DCB angiography; if, after pretreatment, the operator deems rescue stenting necessary, the dissection degree is determined from the angiography taken before stenting. For analysis purposes, lesions with no dissection or classified as Type A/B are grouped into the "nonsevere dissection group," and those classified as Type C/D/E/F are grouped into the "severe dissection group" (Figure 1). Supplemental Figure 2 provides representative images of various types of dissections observed in this study.

### RWS quantification and lesion assessment

RWS analysis was performed using baseline angiography acquired before any predilation or interventional manipulation at an independent core laboratory (cardiovascular interventional diagnosis and treatment image analysis core laboratory platform, Fuwai Hospital, Beijing, China) by 2 certified analysts blinded to procedural outcomes and clinical data. Utilizing AngioPlus Core software (v3.0, Pulse Medical) with validated strain analysis algorithms, the assessment protocol comprised 3 standardized phases: 1) The software's electrocardiogram-gated algorithm automatically identified 4 key cardiac phases: end-diastole, early-systole, end-systole, and mid-diastole. Spatial-temporal registration ensured anatomical correspondence across phases. 2) Within the target lesion segment, the region of interest (ROI) was automatically defined across all phases. Advanced edge-detection algorithms traced lumen contours, followed by manual verification and adjustment by analysts. 3) RWS was computed at each longitudinal position along the angiographic centerline of the interrogated vessel. The RWS value at a given position was calculated as the difference between the maximum and minimum lumen diameters during the cardiac cycle, divided by the maximum lumen diameter at that position. This generated a spatial distribution of RWS values along the lesion segment. The maximum RWS (RWS_max_), representing the highest value among all longitudinal positions within the lesion, was recorded as the final output for analysis. The standard operation procedures were published previously.^15^

**Figure 2:**
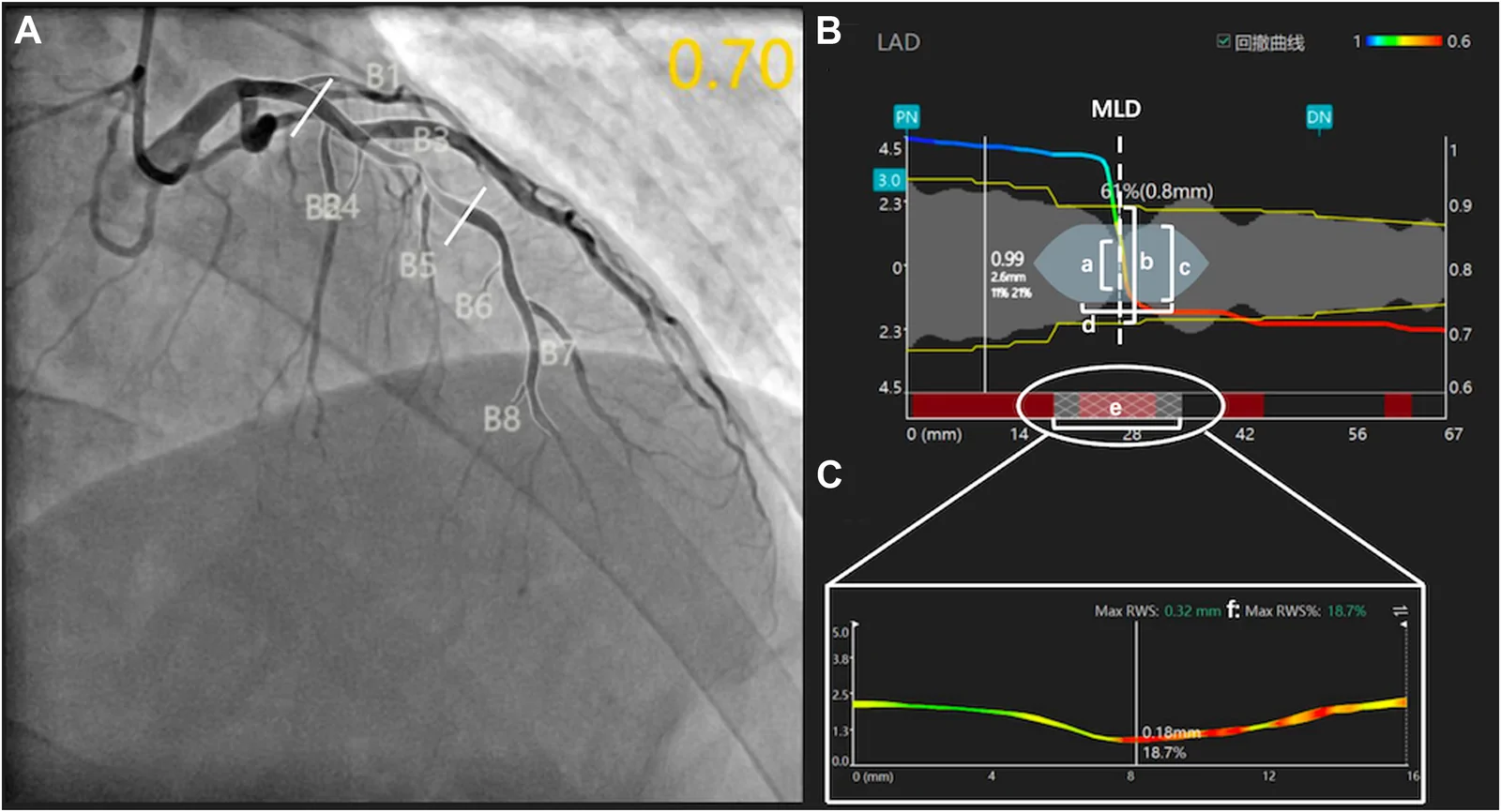
Target Lesion Assessment and RWS Calculation (A) The optimal angiographic view with complete vessel unfolding was selected for analysis. Relatively normal vessel segments proximal and distal to the target lesion were identified as reference segments. (B) The algorithm automatically delineates the ROI, providing the following parameters: a, MLD; b, MLDd; e, lesion length, defined as the automated stent length estimation for conventional PCI planning. For DCB-specific parameters: c, diameter of the final balloon used in DCB strategy; d, balloon length. (C) Following ROI selection, the program computes the RWS at each longitudinal position as the difference between the maximum and the minimum lumen diameters within the cardiac cycle divided by the maximum lumen diameter, and the RWS_max_ is then automatically generated (f). MLD = minimal lumen diameter; MLDd = reference lumen diameter of MLD; PCI = percutaneous coronary intervention; ROI = region of interest; RWS_max_ = maximum radial wall strain; other abbreviations as in Figure 1.

The software's advanced quantification module automatically generated comprehensive lesion characteristics synchronously, including 1) minimal lumen diameter (MLD): the narrowest lumen dimension within the ROI; 2) reference lumen diameter of MLD (MLDd); 3) diameter stenosis (DS%), calculated as 100∗ (MLDd-MLD)/MLDd; 4) area stenosis (AS%), derived from 3-dimensional lumen reconstruction using 100∗(1-MLD^2^/MLDd^2^); 5) lesion length, defined as the automated stent length estimation for conventional PCI planning, determined by the distance between proximal and distal shoulder points where lumen diameter recovers to 90% of MLDd. Figure 2 illustrates the procedural workflow for RWS_max_ analysis and anatomical parameter measurement in a representative case.

### Procedural characteristics of DCB intervention

The type and size of the balloon used for pretreatment were determined by the operator based on the clinical situation. If the operator selected multiple pretreatment balloons, only the type, size, and inflation pressure of the final balloon were recorded in this study, as well as the total number of predilations.

For the DCB procedure, the diameter, length, inflation pressure, and inflation duration of the DCB were recorded. To evaluate the potential impact of DCB size on the degree of dissection, we defined the following ratios: DCB/vessel ratio (defined as 100∗[DCB diameter^2^/MLDd^2^]), DCB/lesion ratio (defined as100∗ DCB diameter^2^/[MLDd^2^∗AS%]), and DCB/lesion length ratio (defined as DCB length divided by lesion length). For patients who underwent only pretreatment followed by rescue stenting, these parameters were calculated using the size of the final pretreatment balloon.

### Statistical analysis

Continuous variables are presented as mean ± SD, and categorical variables as counts (percentages). Intergroup differences were assessed using the chi-square or Fisher's exact test for categorical variables. For continuous variables, comparisons between the 2 groups were made using either the independent samples *t*-test or the Mann-Whitney *U* test, as appropriate based on the data distribution. To evaluate the distribution pattern of RWS_max_ across different dissection grades, we performed linear trend analysis using Jonckheere-Terpstra test to assess monotonic trends across ordered dissection categories. Receiver operating characteristic (ROC) curve analysis was employed to determine the predictive capacity of RWS_max_ for severe dissection, with optimal cutoff values identified using the Youden index. To further enhance the model evaluation, we compared the AUC (area under the curve) of the 2 clinical models and used the DeLong test to assess whether the difference in AUC values between the 2 groups was statistically significant. In addition, net reclassification improvement and integrated discrimination improvement metrics (including 95% CI) were used to quantitatively assess the improvement of the model. Decision curve analysis was performed to assess the clinical net benefit of adding RWSmax to the model across a range of clinically reasonable threshold probabilities.

To further clarify the association between RWS_max_ and severe dissection, we conducted variable selection and multivariable analysis based on the purposeful variable selection method of Hosmer and Lemeshow^16^^,^^17^: All variables with a *P* value <0.20 in the univariable analysis were included in the multivariable analysis 1. Then we gradually eliminated variables with *P* values >0.05 and refitted the model. Finally, covariates that altered the coefficient of the primary variable, RWS_max_, by more than 10% were retained. And variables with a *P* value <0.05 from this analysis were then incorporated into multivariable model 2 for further multivariable analysis (detailed information can be found in Supplemental Tables 1 and 2). To address potential confounding more comprehensively and validate the robustness of our findings, we additionally performed a theory-driven multivariable analysis (model 3). Based on clinical relevance, we included all covariates that are established risk factors for coronary dissection or are known to influence vessel response to balloon injury. To evaluate the incremental value of RWSmax, we compared multivariable logistic regression models with and without this biomarker using the likelihood ratio test, Akaike Information Criterion, and Bayesian Information Criterion. Model calibration was assessed using the Hosmer-Lemeshow goodness-of-fit test.

To exclude the potential confounding factors, serial sensitivity analyses were conducted. First cases with significant residual stenosis (diameter stenosis% >30) following DCB treatment were excluded in Sensitivity Analysis 1, then the aforementioned procedures were repeated. In Sensitivity Analysis 2, we excluded patients who underwent immediate rescue stenting after pretreatment. In addition, we conducted a separate Sensitivity Analysis 3 for patients who received rescue stenting directly after pretreatment without DCB treatment, repeating the same process to investigate the impact of RWS_max_ on dissection severity during the pretreatment phase of the DCB strategy group (Figure 1).

To elucidate the consistency of the association between RWS_max_ and varying degrees of dissection across different predefined subgroups, we established subgroups based on factors such as age, sex, diabetes status, reference vessel diameter, presence of multivessel disease, and target lesion location. In addition, because baseline data revealed that patients in the severe dissection group had a higher prevalence of Type B2/C lesions, severe angulation, severe calcification, and longer lesion lengths, corresponding subgroups were also established. Moreover, due to limited experience in managing NSVL during the early phase of DCB use, we divided the patients into 2 subgroups based on their enrollment time in the study to investigate whether the association between RWS_max_ and dissection severity remains independent of the experience with DCB treatment. To formally test for effect modification, multiplicative interaction terms (eg, RWS_max_ ∼ × subgroup variable) were introduced into the multivariable logistic regression model.

All statistical analyses were performed using R version 4.2.2, and a 2-tailed *P* < 0.05 was considered statistically significant.

## Results

### Patient baseline characteristics

After excluding 6 patients for poor angiography, a total of 194 patients were included in the final analysis. The average age was 57.18 ± 9.62 years, with 72.2% (140 of 194) being male. The distribution of target vessels was as follows: left anterior descending 66.5% (n = 129 of 194), left circumflex 15.5% (n = 30 of 194), and right coronary artery 18.0% (n = 35 of 194). Of these patients, 132 (68.0%) were classified as having no severe dissection, with 35, 45, and 52 patients presenting with no visible dissection, Type A dissection, and Type B dissection, respectively. The severe dissection group comprised 62 patients (32.0%), among whom 83.9% (n = 52) were Type C. In addition, 6 patients exhibited Type D dissection, 3 had Type E dissection, and 1 had a severe Type F dissection accompanied by vessel occlusion (Figure 1).

There were no significant differences between the 2 groups in terms of demographic characteristics such as age, sex, body mass index, and smoking history, nor were there notable differences in past medical history (diabetes, hyperlipidemia, previous myocardial infarction, prior PCI) and clinical data (liver and kidney function, blood lipids, C-reactive protein, left ventricular ejection fraction), as shown in Table 1.

**Table 1:** Baseline Demographic and Clinical Characteristics

Variable	All Patients (N = 194)	Nonsevere Dissection (n = 132)	Severe Dissection (n = 62)	*P* Value
Age, y	57.18 (9.62)	57.11 (9.73)	57.33 (9.46)	0.883
Male	140 (72.2)	91 (68.9)	49 (79.0)	0.197
BMI, kg/m^2^	25.39 [23.66-27.55]	25.43 [23.79-27.55]	25.35 [23.48-27.16]	0.523
Cigarette smoking				0.178
Current smoker	55 (28.4)	32 (24.2)	23 (37.1)	
Former smoker	31 (16.0)	22 (16.7)	9 (14.5)	
Never smoked	108 (55.7)	78 (59.1)	30 (48.4)	
Diabetes	56 (28.9)	37 (28.0)	19 (30.6)	0.838
Hypertension	122 (62.9)	84 (63.6)	38 (61.3)	0.876
Hyperlipidemia	137 (70.6)	95 (72.0)	42 (67.7)	0.664
Previous myocardial infarction	16 (8.2)	7 (5.3)	9 (14.5)	0.058
Previous percutaneous coronary intervention	20 (10.3)	11 (8.3)	9 (14.5)	0.286
Previous stroke	22 (11.3)	14 (10.6)	8 (12.9)	0.820
Chronic kidney disease	2 (1.0)	1 (0.8)	1 (1.6)	1.000
Biochemical test				
Hb, g/L	147.94 (13.58)	146.89 (14.29)	150.16 (11.73)	0.094
Plt, 10^9^/L	217 [190-258]	224 [190-261]	211 [191-253]	0.577
Alb, g/L	46.15 [44.02-48.10]	46.20 [44.00-48.10]	46.10 [44.20-48.70]	0.362
Cr, μmol/L	64.65 [56.82-71.95]	64.31 [57.60-70.55]	65.40 [54.40-74.15]	0.435
LDL, mmol/L	2.20 [1.70-2.84]	2.23 [1.75-2.93]	2.14 [1.56-2.77]	0.349
NT-proBNP, pg/mL	45.05 [17.65-93.55]	47.85 [20.48-97.65]	37.30 [14.10-79.93]	0.239
CRP, mg/L	0.66 [0.23-1.65]	0.65 [0.23-1.56]	0.75 [0.25-1.93]	0.661
Glucose, mmol/L	6.06 [5.33-7.21]	6.04 [5.33-7.29]	6.08 [5.31-6.99]	0.964
TTE				
LVEF, %	65.00 [62.00-67.00]	65.00 [62.00-67.00]	65.00 [62.00-67.75]	0.888

Data are n (%) or median (IQR), except where otherwise stated.

Alb = albumin; BMI = body mass index; Cr = creatinine; CRP = C-reactive protein; Hb = hemoglobin; LDL = low-density lipoprotein; LVEF = left ventricular ejection fraction; NT-pro-BNP = N-terminal pro B-type natriuretic peptide; TTE = transthoracic echocardiology.

Continuous variables are presented as mean (SD) or median [interquartile range].

Assessment of lesion characteristics indicated that the severe dissection group had a higher proportion of patients with Type B2/C lesions (77.4% [48 of 62] vs 50.4% [66 of 132], *P* = 0.001) and severe angulation (21.0% [13 of 62] vs 7.6% [10 of 132], *P* = 0.015), as well as longer target lesions (31.60 [24.29-42.04] mm vs 23.36 [17.73-34.29] mm, *P* < 0.001) and a higher RWS_max_ (14.70% [12.80-16.65] vs 12.20% [11.00-13.30], *P* < 0.001). However, there were no differences between the groups in terms of MLD, MLDd, or degree of lesion stenosis. DCB procedural data showed that there were no significant differences in pretreatment between the 2 groups. The sizes of the DCB and the ratio of the DCB to the lesion's cross-sectional area were similar. Nonetheless, the severe dissection group exhibited a significantly lower DCB/lesion length ratio (57.15% [38.90-82.36] vs 87.03% [56.51-108.66], *P* = 0.001) and a shorter DCB inflation duration (14.00 [7.00-60.00] seconds vs 60.00 [60.00-60.00] seconds, *P* < 0.001) (Table 2).

**Table 2:** Lesion Assessment and Procedural Characteristics

Variable	All Patients (N = 194)	Nonsevere Dissection (n = 132)	Severe Dissection (n = 62)	*P* Value
Lesion characteristics				
Target lesion				0.542
Left anterior descending	129 (66.5)	86 (65.2)	43 (69.4)	
Left circumflex	30 (15.5)	23 (17.4)	7 (11.3)	
Right coronary artery	35 (18.0)	23 (17.4)	12 (19.4)	
Pre TIMI flow grade ≥3	173 (89.6)	115 (87.8)	58 (93.5)	0.428
Type B2/C	114 (59.1)	66 (50.4)	48 (77.4)	**0.001**
Severe angulation	23 (11.9)	10 (7.6)	13 (21.0)	**0.015**
Severe calcification	13 (6.7)	7 (5.3)	6 (9.7)	0.416
Eccentric	179 (92.7)	119 (90.8)	60 (96.8)	0.316
ROI characteristics				
MLD, mm	1.27 [1.07-1.50]	1.27 [1.07-1.53]	1.26 [1.06-1.45]	0.774
Reference lumen diameter of MLD, mm	2.99 (0.51)	2.99 (0.53)	3.00 (0.48)	0.927
Diameter stenosis, %	57.34 [49.73-63.67]	57.05 [48.50-63.39]	58.05 [51.37-63.81]	0.484
Area stenosis, %	81.80 [74.73-86.80]	81.55 [73.47-86.60]	82.40 [76.35-86.90]	0.484
Lesion length, mm	26.69 [18.74-36.13]	23.36 [17.73-34.29]	31.60 [24.29-42.04]	**<0.001**
RWS_max_, %	12.60 [11.30-14.57]	12.20 [11.00-13.30]	14.70 [12.80-16.65]	**<0.001**
Procedural characteristics				
Transradial intervention	188 (96.9)	128 (97.0)	60 (96.8)	1.000
Lesion pretreatment before DCB				
Type of predilation balloon				0.371
Cutting or scoring balloon	175 (90.2)	121 (91.7)	54 (87.1)	
Noncompliant balloon	16 (8.2)	10 (7.6)	6 (9.7)	
Semicompliant balloon	3 (1.5)	1 (0.8)	2 (3.2)	
No. of predilation	2.00 [2.00-3.00]	2.00 [2.00-3.00]	3.00 [2.00-3.00]	0.213
Final predilation balloon diameter, mm	3.00 [2.75-3.00]	3.00 [2.75-3.00]	3.00 [2.75-3.00]	0.427
Final predilation balloon length, mm	10.00 [10.00-13.00]	10.00 [10.00-13.00]	10.00 [10.00-13.00]	0.561
Max predilation pressure, atm	12.00 [10.00-14.00]	12.00 [10.00-14.00]	12.00 [10.00-14.00]	0.867
DCB treatment				
DCB diameter, mm	3.00 [2.7.5-3.00]	3.00 [2.75-3.00]	3.00 [2.75-3.00]	0.265
DCB length, mm	20.00 [15.00-25.00]	20.00 [15.00-21.25]	17.50 [13.00-25.00]	0.249
DCB/artery ratio, %	106.2 [81.6-125.2]	107.5 [84.0-130.2]	99.3 [76.6-120.5]	0.115
DCB/lesion ratio, %	130.9 [102.9-165.2]	133.6 [108.7-165.8]	127.0 [91.9-163.1]	0.243
DCB/length ratio, %	73.94 [47.41-104.07]	87.03 [56.51-108.66]	57.15 [38.90-82.36]	**0.001**
DCB inflation pressure, atm	10.00 [8.00-12.00]	10.00 [8.00-12.00]	12.00 [8.00-14.00]	0.160
DCB inflation duration, s	60.00 [9.25-60.00]	60.00 [60.00-60.00]	14.00 [7.00-60.00]	**<0.001**
Fluoroscopy time, min	23.00 [16.00-32.00]	21.00 [15.00-29.25]	30.00 [20.00-37.75]	**<0.001**
Contrast medium used, mL	170 [160-180]	170 [150-180]	170 [160-180]	0.554

Data are n (%) or median (IQR), except where otherwise stated.

DCB = drug-coated balloon; MLD = minimum lumen diameter; ROI = region of interest; RWS_max_ = maximum radial wall strain; TIMI = Thrombolysis In Myocardial Infarction.

Continuous variables are presented as mean (SD) or median [interquartile range]. *P* values <0.05 are shown in **bold**.

### Value of RWS_max_ in predicting the severity of coronary dissection

There was an upward trend in RWS_max_ as the severity of dissection increased. Specifically, the average RWS_max_ was 11.5% in 35 patients with no dissection; 12.1% in 45 patients with Type A dissection; 13.0% in 52 patients with Type B dissection; 14.7% in 52 patients with Type C dissection; and RWSmax reached 15.4% in the 10 patients with Type D/E/F dissections (Jonckheere-Terpstra *P* < 0.001) (Supplemental Figure 3).

**Figure 3:**
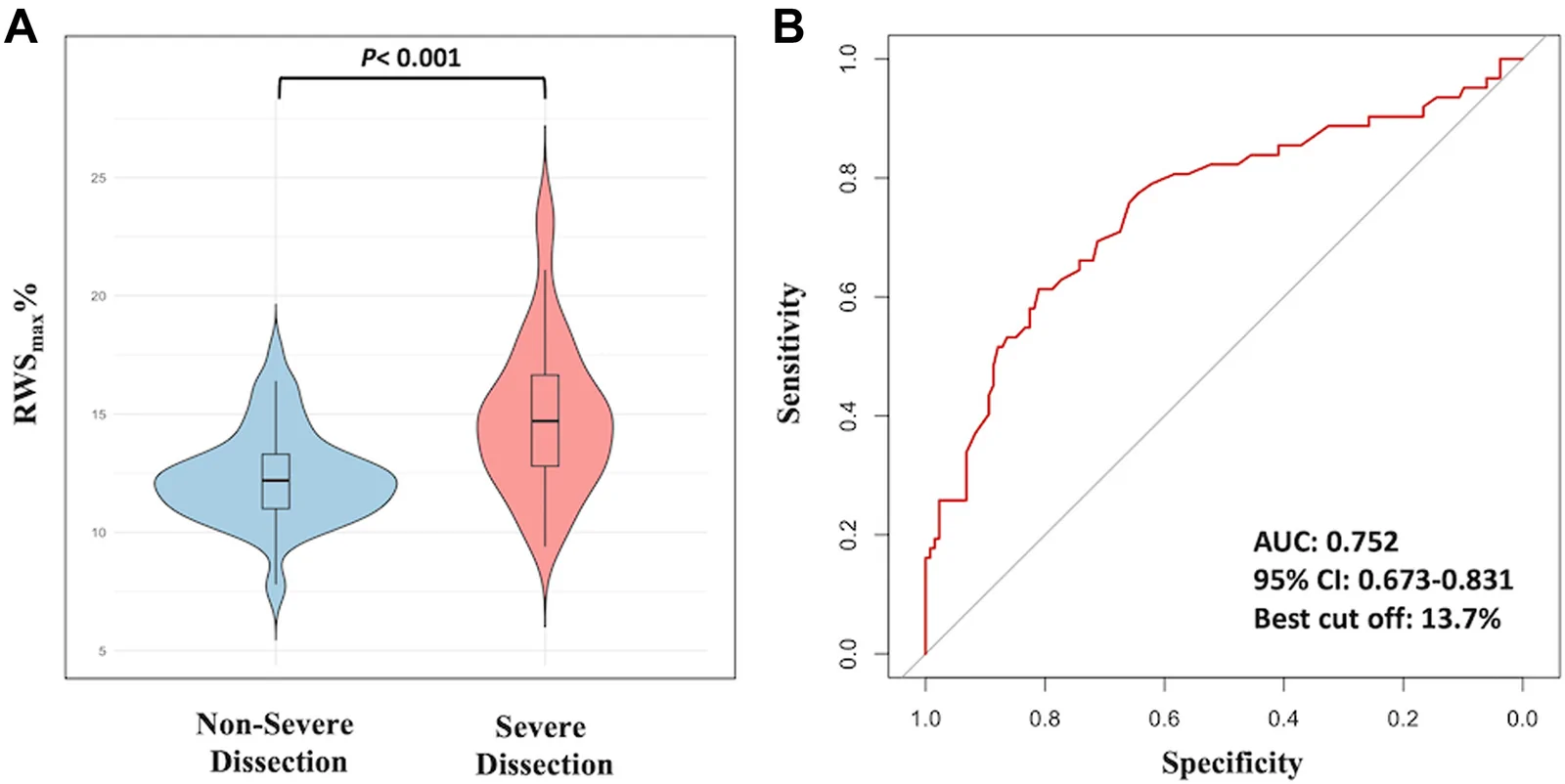
RWS_max_ as a Predictor of Severe Dissection (A) Comparison of RWS_max_ between groups was performed using independent samples *t*-test (*P* < 0.001). Patients with severe dissection showed significantly higher RWS_max_ values. (B) ROC curve analysis for RWS_max_ in predicting severe dissection after DCB treatment in non–small vessel lesions. RWS_max_ demonstrated good predictive performance with an AUC of 0.752 (95% CI: 0.673-0.831). The optimal cutoff value of RWS_max_ for predicting severe dissection was 13.7%. AUC = area under the curve; ROC = receiver operating characteristic; other abbreviations as in Figures 1 and 2.

Furthermore, as shown in Figure 3A, the average RWS_max_ in the severe dissection group was significantly higher than that in the nonsevere group (14.8% [3.20] vs 12.3% [1.94], *P* < 0.001), and ROC analysis indicated that RWS_max_ has good predictive ability for severe dissection, with an AUC of 0.752 (95% CI: 0.673-0.831). The optimal cutoff value was determined to be 13.7%, yielding a sensitivity of 61.3% [56.5%-66.1%] and a specificity of 81.1% [74.4%-87.8%] (Figure 3B).

**Figure 4:**
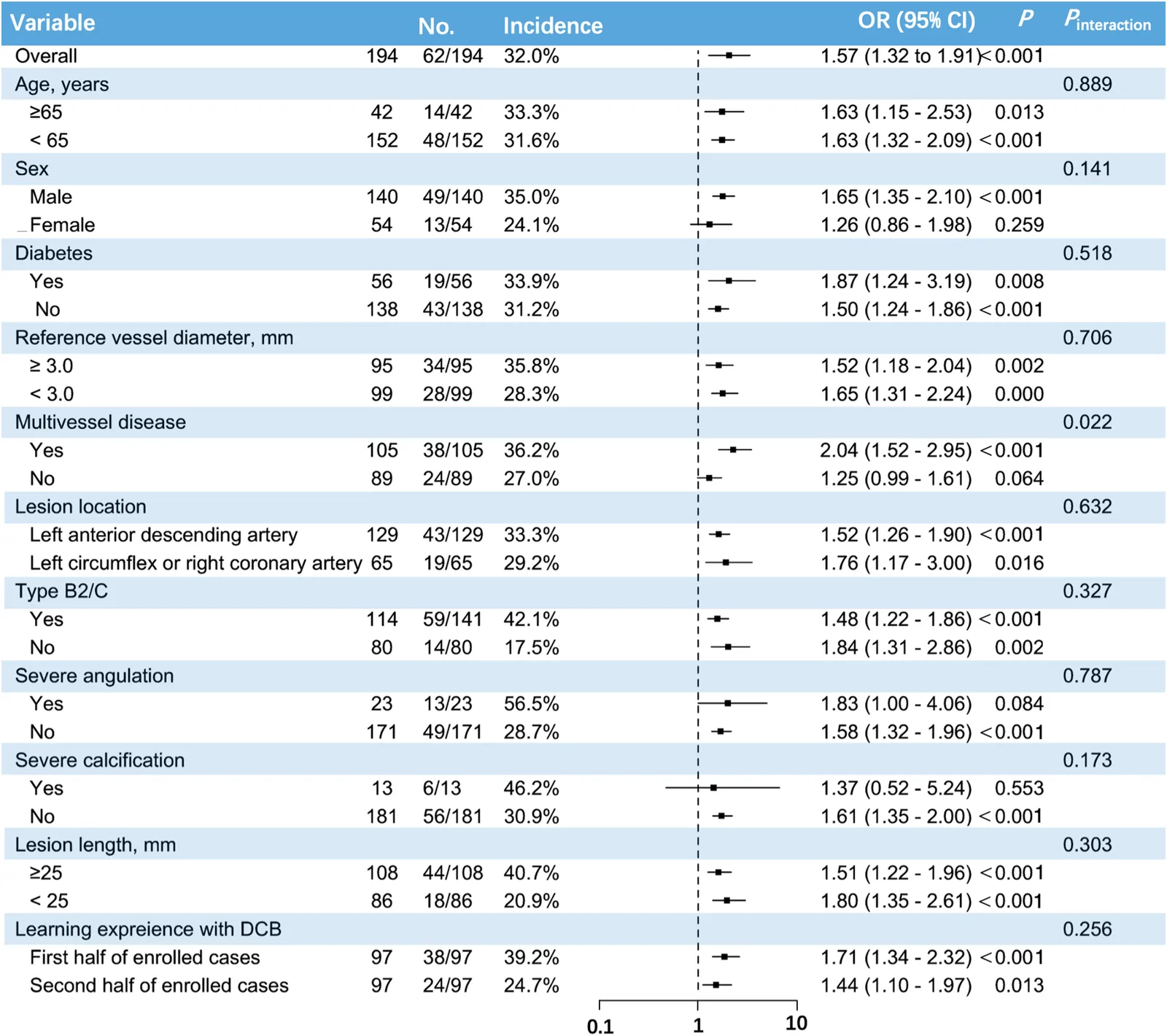
Subgroup Analyses for Severe Dissection The forest plot displays ORs with 95% CIs, adjusted for Type B2/C, Severe angulation, RWS_max_, DCB/length ratio, and DCB inflation duration. *P* values for subgroup comparisons were derived from multivariable logistic regression and represent interaction effects between subgroups. Abbreviations as in Figures 1 and 2.

**Figure 5:**
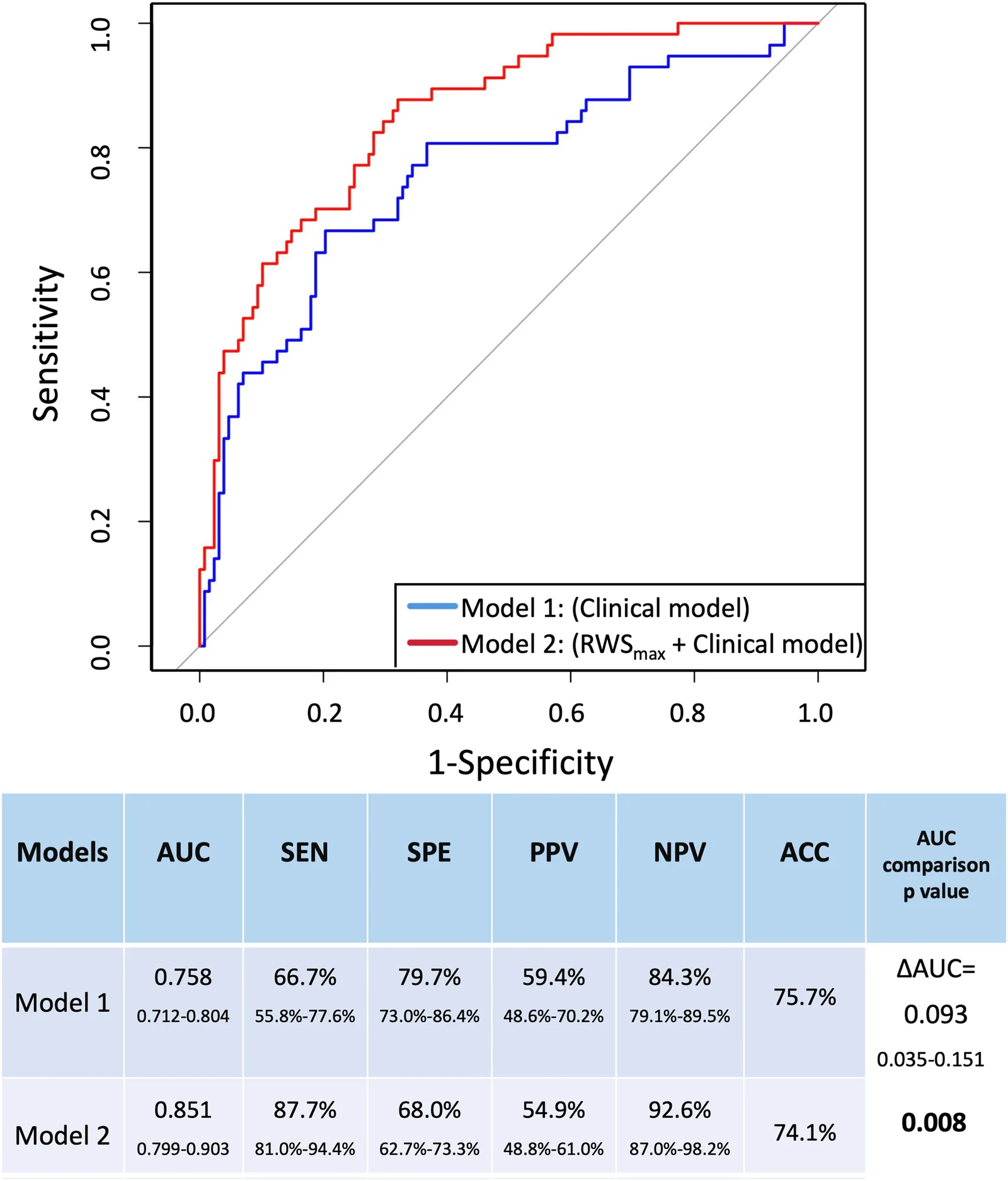
Incremental Prognostic Value of RWS_max_ in Predicting Severe Dissection After DCB Strategy Comparison of receiver operating characteristic curves was performed using DeLong's test with *P* = 0.008. ACC = accuracy; NPV = negative predictive value; PPV = positive predictive value; SEN = sensitivity; SPE = specificity; other abbreviations as in Figures 2 and 3.

In sensitivity analyses, RWS_max_ consistently demonstrated good predictive ability for severe dissection across different populations. In sensitivity analysis 1 (n = 184), the severe dissection group still exhibited a significantly higher RWS_max_ compared with the nonsevere group (14.70% [12.80-16.65] vs 12.20% [11.00-13.30], *P* < 0.001), with an AUC of 0.742 and an optimal cutoff of 13.7%. In sensitivity analysis 2 (n = 160), the optimal cutoff was 12.65%, whereas in sensitivity analysis 3 (n = 34), the optimal cutoff for predicting severe dissection was 14.3% (see Supplemental Tables 3 to 5, Figures 4 and 5, Central Illustration).

**Central Illustration:**
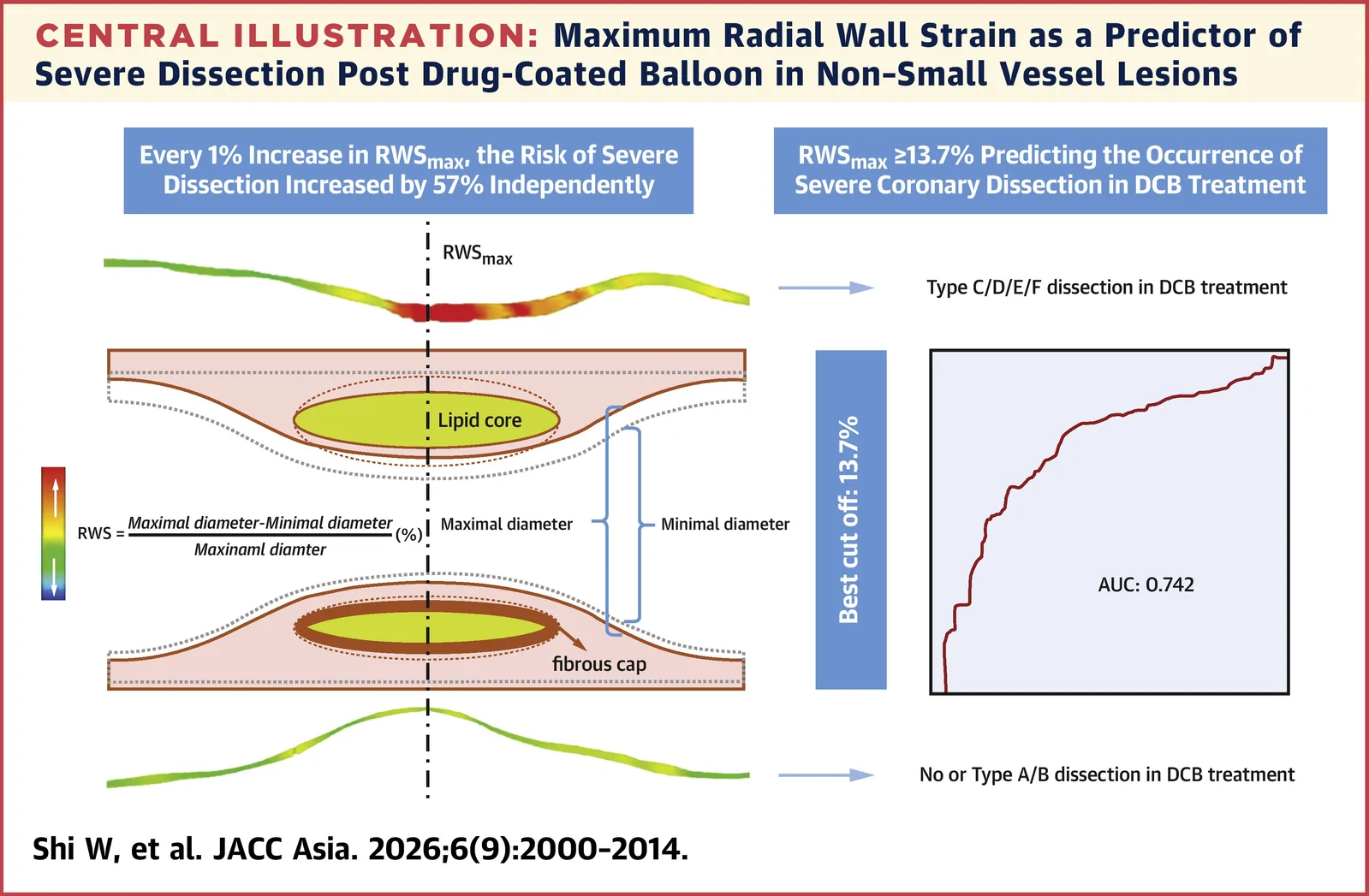
Maximum Radial Wall Strain as a Predictor of Severe Dissection Post Drug-Coated Balloon in Non–Small Vessel Lesions A target vessel angiographic image from a single cardiac cycle was acquired, and after selecting the region of interest, the RWS of each point was automatically generated, with the RWS_max_ output. RWS_max_ is associated with plaque characteristics—plaques with larger lipid core volume and thinner fibrous caps tend to have higher RWS_max_, making it a potential indicator for assessing plaque vulnerability. Receiver operating characteristic analysis identified 13.7% as the optimal cutoff value of RWS_max_ for predicting severe dissection (Type C or higher) following DCB treatment in non–small vessel lesions (≥2.75 mm). Furthermore, multivariate analysis indicated that for every 1% increase in RWS_max_, the risk of severe dissection increased by 57% (OR: 1.57; 95% CI: 1.32-1.91; *P* < 0.001). DCB = drug-coated balloon; RWS = radial wall strain; RWS_max_ = maximum radial wall strain.

### RWS_max_ as an independent risk factor for severe coronary dissection

We conducted multivariable analysis to further clarify that RWS_max_ is an independent predictor of severe coronary dissection. In multivariable model 1, which included variables significant in the univariable analysis, the results showed that for every 1% increase in RWS_max_, the risk of severe dissection increased by 55% (OR: 1.55; 95% CI: 1.31-1.90; *P* < 0.001), independent of other clinical or procedural factors (Table 3). Further, when we included the significant variables from model 1 into model 2, RWS_max_ remained an independent risk factor for severe dissection in the DCB strategy group (OR: 1.57; 95% CI: 1.32-1.91; *P* < 0.001) (Table 3).

**Table 3:** Predictors of Severe Dissection in the Overall Population by Multivariable Regression Analysis

	Multivariable Analysis 1		Multivariable Analysis 2		Multivariable Analysis 3	
	OR (95% CI)	*P* Value	OR (95% CI)	*P* Value	OR (95% CI)	*P* Value
Age					0.88 (0.53-1.43)	0.603
Male	1.37 (0.54-3.71)	0.516			0.79 (0.25-2.48)	0.682
Smoker					1.42 (0.56-3.62)	0.463
Diabetes					0.91 (0.32-2.47)	0.850
Hypertension					0.42 (0.15-1.12)	0.088
Hyperlipidemia					0.61 (0.22-1.65)	0.326
Previous myocardial infarction	2.90 (0.44-24.35)	0.288			3.77 (0.45-35.07)	0.224
Previous PCI	1.25 (0.14-8.22)	0.825			1.50 (0.14-14.25)	0.733
Type B2/C	**2.60 (1.06-6.79)**	**0.041**	1.45 (1.00-5.47)	0.055	1.60 (0.56-4.74)	0.384
Severe angulation	**3.59 (1.11-12.23)**	**0.022**	**3.62 (1.17-11.76)**	**0.027**	**4.79 (1.34-18.46)**	**0.018**
RWS_max_, (1% change)	**1.55 (1.31-1.90)**	**<0.001**	**1.57 (1.32-1.91)**	**<0.001**	**1.77 (1.42-2.31)**	**<0.001**
No. of predilation	1.33 (0.65-2.73)	0.437			1.30 (0.95-1.81)	0.110
DCB/length ratio (1% change)	**0.99 (0.98-1.00)**	**0.070**	**0.99 (0.98-1.00)**	**0.044**	0.99 (0.98-1.00)	0.125
DCB inflation pressure, (1 atm change)	1.04 (0.90-1.19)	0.613			1.02 (0.88-1.18)	0.805
DCB inflation duration, (1s change)	**0.98 (0.97-1.00)**	**0.019**	**0.98 (0.97-0.99)**	**0.003**	**0.98 (0.96-0.99)**	**0.010**

*P* values <0.05 are shown in **bold**.

PCI = percutaneous coronary intervention; other abbreviations as in Table 2.

To address potential confounding more comprehensively, we constructed a theory-driven model 3 that included all clinically relevant covariates based on literature review and clinical knowledge. In this model, RWS_max_ remained a robust independent predictor of severe dissection (OR: 1.77; 95% CI: 1.42-2.31; *P* < 0.001). To evaluate the incremental value of RWS_max_, we compared model performance with and without this biomarker. The addition of RWS_max_ significantly improved model fit, as evidenced by lower Akaike Information Criterion and Bayesian Information Criterion values and improved calibration (Hosmer-Lemeshow test: *P* = 0.277 vs *P* = 0.076 without RWS_max_). Detailed model performance comparisons are presented in Supplemental Table 6.

Additional sensitivity analyses supported this conclusion (Supplemental Table 7). In sensitivity analyses 1and 2, we found that for every 1% increase in RWS_max_, the risk of severe dissection increased by 55% (OR: 1.54; 95% CI: 1.30-1.88; *P* < 0.001) and 60% (OR: 1.60; 95% CI: 1.31-2.02; *P* < 0.001), respectively. However, in sensitivity analysis 3, the difference did not reach statistical significance (OR: 1.33; 95% CI: 0.95-2.04; *P* = 0.131).

### Subgroup analysis

As shown in Figure 4, the multivariable results in the subgroups based on age, sex, diabetes status, reference vessel diameter, and target lesion location were consistent with the results from the overall population. Analyses based on different target lesion morphological subgroups indicated that the correlation between RWS_max_ and dissection severity was not affected by factors such as severe angulation, lesion type, calcification, or lesion length. However, for every 1% increase in RWS_max_, the correlation with severe dissection appeared to be more pronounced in patients with multivessel disease (*P* for interaction = 0.022). Given that later operators recognized the importance of choosing relatively smaller pretreatment balloons and using a “slow up slow down and wait” approach for NSVL DCB treatments, we also performed a subgroup analysis based on patient enrollment sequence. The results showed no significant differences between the groups (*P* for interaction = 0.256).

### Incremental prognostic value of RWS_max_ in predicting severe dissection in DCB strategy

Based on multivariable analysis, we developed a clinical model incorporating target lesion characteristics and procedural features to predict the severity of dissection (AUC = 0.758 [0.712-0.804]). When RWS_max_ was added to this model, the predictive capability significantly improved (AUC = 0.851 [0.799-0.903], DeLong test *P* = 0.008),with a corresponding continuous net reclassification improvement of 0.819 (95% CI: 0.533-1.094) and integrated discrimination improvement of 0.147 (95% CI: 0.088-0.203) (Figure 5 and Supplemental Figure 7). Decision curve analysis further demonstrated that the model incorporating RWS_max_ provided consistently higher net benefit across a wide range of clinically relevant threshold probabilities compared with the model without RWS_max_ (Supplemental Figure 8), confirming its clinical utility. This underscores the incremental value of biomechanical plaque assessment in risk stratification.

## Discussion

RWS_max_ is associated with the degree of coronary dissection after balloon dilation in the DCB strategy. Even after adjusting for balloon size, inflation pressure, and coronary anatomical features, RWS_max_ remains independently associated with dissection severity. An RWS_max_ value of 13.7% is the optimal cutoff for predicting the occurrence of severe coronary dissection (Central Illustration). When RWS_max_ was incorporated into the severe dissection prediction model based on target lesion anatomical characteristics, the overall predictive ability of the model significantly improved. This suggests that in clinical practice, the application of RWS_max_ can better identify patients who may develop severe dissection after receiving the DCB strategy. This provides a potential direction for future research in selecting patients suitable for DCB treatment in primary NSVL.

Severe coronary dissections not only lead to DCB strategy failure requiring rescue stenting,^18^^,^^19^ but may also precipitate acute lumen loss and periprocedural major adverse cardiovascular events if untreated.^20^ This underscores the critical importance of preprocedural lesion selection for DCB therapy. RWS_max_ represents an innovative, angiography-based computational algorithm that enables plaque characterization without additional intracoronary manipulation.^21^ Validated by intravascular imaging studies, RWS exhibits strong correlations with high-risk plaque characteristics, including larger lipid-rich necrotic cores and thinner fibrous caps, both of which predispose to plaque progression and acute coronary syndromes.^15^^,^^22^ Moreover, emerging evidence demonstrates that RWS_max_ effectively identifies high-risk vulnerable plaques in carotid arteries,^23^ precisely reflecting the biomechanical properties of atherosclerotic lesions. These findings underscore RWS technology's capacity to characterize pan-vascular plaque vulnerability, demonstrating its broad application potential across vascular territories. However, no study to date has elucidated its relationship with immediate procedural outcomes following DCB treatment. According to our results, RWS_max_ is closely associated with the degree of dissection, even after adjusting for procedural factors and lesion characteristics, indicating that RWS_max_ can be used to identify high-risk patients who may develop severe dissection. This technique may be applied in clinical practice in the future.

Through multivariate analysis, we observed that the anatomical characteristics of target lesions were significantly associated with severe dissection, such as Type B2/C, severe angulation, and others. In addition, we noticed that the lower the DCB/lesion length ratio, the higher the risk of severe dissection. Previous studies have also suggested that longer lesions are related to dissection.^19^^,^^20^ This suggests that in the DCB strategy, choosing a shorter balloon that does not fully cover the lesion may cause dissection due to plaque compression, displacement, or other factors. Therefore, selecting a longer DCB to cover the lesion as much as possible should be considered. In line with previous study,^19^ our findings indicate a negative correlation between balloon dilation time and the severity of dissections. Longer dilation times may allow the dissection flap to better appose against the vessel wall, potentially reducing the extent of macroscopic dissection observed.

Further, based on the preceding findings, we developed a model for predicting severe dissection. We found that introducing RWS_max_ significantly improved the predictive ability, further indicating that the existing anatomical classifications are insufficient for screening patients suitable for DCB treatment. Routine evaluation of RWS_max_ should be considered in clinical practice.

Considering that multivariate analysis indicated a significant relationship between the anatomical characteristics of the target lesions and severe dissection, subgroup analysis based on whether the lesions were Type B2/C, whether they had severe angulation, severe calcification, or lesion length revealed that RWS_max_ was independently associated with severe dissection in target lesions with various anatomical features. However, when we stratified patients by the presence of multivessel disease, the association between RWS_max_ and severe dissection was even stronger in those with multivessel involvement. This may be because patients with multiple lesions carry a heavier lipid burden and have more unstable plaques, and previous studies have shown that these patients are more likely to experience long-term progression.^24^ These findings suggest that, in patients with visually assessed stenosis less than 70%, RWS_max_ analysis should be performed routinely so that individuals who are unsuitable for a DCB strategy can be identified and excluded.

Our experience with DCB in NSVL was limited in the early stages. The operators tended to use larger balloons to achieve optimal lesion reconstruction, and the expansion pressure were often higher. As a result, the incidence of severe dissection in the early enrolled patients reached 39.2%. However, as the operators gained more experience, the DCB procedures became gentler, and the balloon inflation strategy evolved to a “slow up slow down and wait” approach. Consequently, the overall incidence of severe dissection decreased to 24.7%. Subgroup analysis showed that RWS_max_ demonstrated predictive ability for severe dissection in both patient groups, with no significant interaction observed. This suggests that RWS_max_ reflects the lesion characteristics and that its relationship with severe dissection is not influenced by the operational factors. Therefore, even in patients who undergo gentler procedures with smaller device sizes, those with higher RWS_max_ still face a higher risk of severe dissection due to plaque rupture.

Supplemental Figures 9 to 12 present detailed procedural data and RWS_max_ analyses for 4 representative cases treated with DCB, with OCT validation available for selected cases. Despite severe baseline stenosis, Cases A and B (with RWS_max_ values of 9.9% and 7.2%, respectively) demonstrated no significant dissection following adequate lesion pretreatment and subsequent DCB therapy. Notably, OCT imaging in case B revealed absence of substantial lipid core with fibrous cap thickness in the ROI, providing direct morphological evidence supporting our central hypothesis that RWS_max_ correlates with plaque vulnerability and consequently predicts dissection risk post-DCB. In contrast, Cases C and D developed Type D dissections requiring rescue stenting despite standardized treatment protocols, attributable to their elevated RWS_max_ values (21.0% and 15.0%, respectively). This case series establishes RWS_max_ quantification as a novel risk-stratification tool in the DCB era, enabling operators to identify high-risk dissection candidates or optimize therapeutic strategies through tailored lesion preparation intensity or proactive stent backup planning.

### Study limitations

Although our study provides a potential direction for optimizing the application of DCB strategy in NSVL, there are several limitations that should be noted: 1) Although previous studies have suggested that RWS_max_ is correlated with plaque characteristics, and our study confirms the association between RWS_max_ and severe dissection, there is still a lack of direct evidence linking plaque instability with severe dissection. Moreover, our assessment of dissection severity was based on angiography. Future studies should incorporate intracoronary imaging to complete the evidence chain of “plaque characteristics—RWS_max_—accurate dissection severity.” 2) This study only focused on the correlation between RWS_max_ and immediate postprocedural outcomes. Therefore, the association between acute clinical event or long-term prognosis with RWS_max_ in NSVL treated with DCB remains unclear. 3) This is a single-center, small sample study. Although subgroup analysis suggests that RWS_max_'s impact on severe dissection is not influenced by lesion anatomy, operator experience, and other factors, caution should be exercised when generalizing these findings. 4) The accuracy of RWS measurement is inherently dependent on the quality of baseline angiography, as precise lumen contour detection is fundamental to strain calculation. In our study, 6 patients were excluded from the analysis due to suboptimal angiographic image quality (eg, vessel overlap, foreshortening, or insufficient contrast opacification), highlighting this technical limitation.

## Conclusions

This study demonstrates that RWS_max_, a novel angiographically derived parameter, serves as an independent predictor of severe coronary dissection following DCB strategy in de novo non–small vessel lesions.

## Funding Support and Author Disclosures

This study was supported by CAMS Innovation Fund for Medical Sciences (CIFMS) (2022-I2M-C&T-B-042) and National High Level Hospital Clinical Research Funding (2022-GSP-QN-2 and 2025-GSP-QN-30). The authors have reported that they have no relationships relevant to the contents of this paper to disclose.
