# Supplementary material for: Radial Wall Strain for Predicting Severe Dissection After Drug-Coated Balloon
Source: JACC Asia. 2026 Jul 18;6(9):2000–14. doi: 10.1016/j.jacasi.2026.05.035 (PMC13554768; doi:10.1016/j.jacasi.2026.05.035)
Supplement: Supplementary file 1 [file mmc1.docx]

**Supplemental Appendix**

**Association Between Angiographically Derived Radial Wall Strain and the Severity of Coronary Dissection After Drug-Coated Balloon Angioplasty in Patients With De Novo Large Coronary Artery Lesions**

**INDEX**

Supplemental Figure 1. STARD Statement	2

Supplemental Figure 2. Representative Angiographic Outcome of Dissections Left After DCB Angioplasty	3

Supplemental Figure 3. Trend of RWS_max_ Across Different Dissection Severities	4

Supplemental Figure 4. Differences in RWS_max_ Between Patients With or Without Severe Dissection (A), and ROC Analysis for RWS_max_ in Predicting Severe Dissection After Drug-Coated Balloon Angioplasty in Patients After Excluding Those With Significant Residual Stenosis (B)	5

Supplemental Figure 5. Differences in RWS_max_ Between Patients With or Without Severe Dissection (A), and ROC Analysis for RWS_max_ in Predicting Severe Dissection After Drug-Coated Balloon Angioplasty in Patients After Excluding Those With Immediate Rescue Stenting After Pre-Treatment (B)	6

Supplemental Figure 6. Differences in Differences in RWS_max_ Between Patients With or Without Severe Dissection (A), and ROC Analysis for RWS_max_ in Predicting Severe Dissection After Drug-Coated Balloon Angioplasty in Patients Undergoing Immediate Rescue Stenting After Pre-Treatment (B)	7

Supplemental Figure 7. Comparison of Predictive Performance Between the Novel Model and Baseline Model	8

Supplemental Figure 8. Decision Curve Analysis for Predicting Severe Dissection	9

Supplemental Figure 9. Representative Case A--- Low RWS_max_ with No dissection	10

Supplemental Figure 10. Representative Case B--- Low RWS_max_ with No dissection (OCT showed absence of substantial lipid core with fibrous cap thickness >120μm in the ROI)	11

Supplemental Figure 11. Representative Case C--- High RWS_max_ with Type D dissection	12

Supplemental Figure 12. Representative Case D--- High RWS_max_ with Type D dissection	13

Supplemental Table 1. Univariable Analysis for Variable Selection in Multivariable Model 1	14

Supplemental Table 2. RWS_max_ Coefficient Change Analysis	15

Supplemental Table 3. Baseline Characteristics of Patients in Sensitivity Analysis 1	16

Supplemental Table 4. Baseline Characteristics of Patients in Sensitivity Analysis 2	19

Supplemental Table 5. Baseline Characteristics of Patients in Sensitivity Analysis 3	22

Supplemental Table 6. Model Performance Comparison With and Without RWSmax	25

Supplemental Table 7. Predictors of Severe Dissection by Multivariable Regression Analysis in Sensitivity Analysis	25

**Supplemental Figure 1: STARD Statement**

**Supplemental Figure 2: Representative Angiographic Outcome of Dissections Left After DCB Angioplasty**

A to F represent typical angiographic images of coronary dissection observed after DCB treatment. Among them, dissections D, E, and F occurred after pre-dilation and were managed with rescue stenting without undergoing DCB treatment. In contrast, dissections images of A, B, and C were obtained after adequate pre-dilation followed by DCB treatment.

**Supplemental Figure 3: Trend of RWS_max_ Across Different Dissection Severities**

**Supplemental Figure 4: Differences in RWS_max_ Between Patients With or Without Severe Dissection (A), and ROC Analysis for RWS_max_ in Predicting Severe Dissection After Drug-Coated Balloon Angioplasty in Patients After Excluding Those With Significant Residual Stenosis (B)**

**Supplemental Figure 5: Differences in RWS_max_ Between Patients With or Without Severe Dissection (A), and ROC Analysis for RWS_max_ in Predicting Severe Dissection After Drug-Coated Balloon Angioplasty in Patients After Excluding Those With Immediate Rescue Stenting After Pre-Treatment (B)**

**Supplemental Figure 6: Differences in RWS_max_ Between Patients With or Without Severe Dissection (A), and ROC Analysis for RWS_max_ in Predicting Severe Dissection After Drug-Coated Balloon Angioplasty in Patients Undergoing Immediate Rescue Stenting After Pre-Treatment (B)**

**Supplemental Figure 7: Comparison of Predictive Performance Between the Novel Model and Baseline Model**

The figure shows that the new model incorporating RWS generally increases the predicted probability of severe dissection (shifting to the right of the dashed line), while significantly decreasing the predicted probability for patients without severe dissection (shifting to the left of the dashed line). The NRI is 0.819 (95% CI: 0.533–1.094), and the IDI is 0.147 (95% CI: 0.088–0.203). This indicates that the new model with RWS correctly reclassified 86.3% of the event group patients into the high-risk category and 72.1% of the non-event group patients into the low-risk category, with a net improvement of 81.9%.

**Supplemental Figure 8: Decision Curve Analysis for Predicting Severe Dissection**

Decision curve analysis comparing the net benefit of prediction models with and without RWSmax across a range of threshold probabilities for severe dissection. The red curve represents the model incorporating RWSmax; the blue curve represents the model without RWSmax. The gray dashed line represents the "treat all" strategy, and the black dotted line represents the "treat none" strategy (net benefit = 0). The model with RWSmax demonstrates consistently higher net benefit across all clinically relevant threshold probabilities, indicating its superior clinical utility for guiding intervention decisions.

**Supplemental Figure 9: Representative Case A--- Low RWS_max_ with No dissection**

**Supplemental Figure 10: Representative Case B--- Low RWS_max_ with No dissection (OCT showed absence of substantial lipid core with fibrous cap thickness >120μm in the ROI)**

**Supplemental Figure 11: Representative Case C--- High RWS_max_ with Type D dissection**

**Supplemental Figure 12: Representative Case D--- High RWS_max_ with Type D dissection**

**Supplemental Table 1: Univariable Analysis for Variable Selection in Multivariable Model 1**

|  | **Univariable analysis** | |
| --- | --- | --- |
|  | **Odd Ratio (95% CI)** | ***P* value** |
| Male | **1.70 (0.85 to 3.57)** | **0.146** |
| Cigarette smoking |  |  |
| Current smoker | 1.87 (0.94 to 3.70) | 0.072 |
| Former smoker | 1.06 (0.42 to 2.52) | 0.891 |
| Never smoked | Ref. |  |
| Clinical presentation |  |  |
| Asymptomatic ischemia | Ref. |  |
| Stable angina | 1.72 (0.68 to 4.65) | 0.264 |
| Unstable angina | 1.26 (0.52 to 3.29) | 0.621 |
| Post myocardial infarction (more than 6 months) | / | / |
| Previous myocardial infarction | **3.03 (1.08 to 8.89)** | **0.036** |
| Previous PCI | **1.87 (0.71 to 4.78)** | **0.192** |
| Type B2/C | **3.38 (1.73 to 6.90)** | **0.001** |
| Severe angulation | **3.21 (1.32 to 7.99)** | **0.010** |
| RWS_max_ | **1.52 (1.32 to 1.79)** | **< 0.001** |
| No. of pre-dilation | **1.45 (0.89 to 2.39)** | **0.136** |
| DCB/length ratio | **0.99 (0.98 to 1.00)** | **0.070** |
| DCB inflation pressure, atm | **1.07 (0.98 to 1.18)** | **0.135** |
| DCB inflation duration, s | **0.98 (0.97 to 0.99)** | **< 0.001** |

Variables with a p-value < 0.20 in the baseline comparison between groups were included in the univariable analysis. Subsequently, variables with a p-value < 0.20 in the univariable analysis were entered into Multivariable Analysis 1. CI = Confidence Interval; PCI= Percutaneous Coronary Intervention; RWSmax = maximum Radial Wall Strain; DCB = Drug-Coated Balloon

**Supplemental Table 2: RWS_max_ Coefficient Change Analysis**

| **Variable** | **RWS Coefficient After Removal** | **Change Percentage (%)** |
| --- | --- | --- |
| Male | 1.57 | 0.56 |
| Previous myocardial infarction | 1.57 | 0.31 |
| Previous PCI | 1.56 | 0.08 |
| No. of pre-dilation | 1.58 | 0.64 |
| DCB inflation pressure | 1.57 | 0.03 |

**After removing any non-significant variables (p-values > 0.05) from Multivariable Model 1, the coefficient of** RWS_max_ **did not change by more than 10%. Therefore, these variables were not included in the final Multivariable Model 2. To further evaluate whether there was multicollinearity among the variables in Multivariable Model 2, we conducted a VIF analysis. The results indicated that all VIF values ranged between 1.0 and 1.2, which is well below the threshold of 5, suggesting that Multivariable Model 2 did not exhibit significant multicollinearity issues.**

**Supplemental Table 3: Baseline Characteristics of Patients in Sensitivity Analysis 1**

| **Variable** | **All patients**  **(n=184)** | **Non-Severe Dissection**  **(n=122)** | **Severe Dissection**  **(n=62)** | ***P* value** |
| --- | --- | --- | --- | --- |
| age (y) | 57.22 (9.72) | 57.16 (9.88) | 57.33 (9.46) | 0.915 |
| Male | 133 (72.3) | 84 (68.9) | 49 (79.0) | 0.199 |
| BMI (kg/m2) | 25.76 (3.97) | 25.88 (4.23) | 25.53 (3.42) | 0.566 |
| Cigarette smoking, |  |  |  | 0.165 |
| Current smoker | 52 (28.3) | 29 (23.8) | 23 (37.1) |  |
| Former smoker | 30 (16.3) | 21 (17.2) | 9 (14.5) |  |
| Never smoked | 102 (55.4) | 72 (59.0) | 30 (48.4) |  |
| Clinical presentation |  |  |  | 0.025 |
| Asymptomatic ischemia | 32 (17.5) | 24 (19.7) | 8 (13.1) |  |
| Stable angina | 61 (33.3) | 39 (32.0) | 22 (36.1) |  |
| Unstable angina | 86 (47.0) | 59 (48.4) | 27 (44.3) |  |
| Post myocardial infarction (more than 6 months) | 4 (2.2) | 0 (0.0) | 4 (6.6) |  |
| Diabetes | 56 (30.4) | 37 (30.3) | 19 (30.6) | 1.000 |
| Hypertension | 117 (63.6) | 79 (64.8) | 38 (61.3) | 0.765 |
| Hyperlipidemia | 131 (71.2) | 89 (73.0) | 42 (67.7) | 0.572 |
| Previous myocardial infarction | 16 (8.7) | 7 (5.7) | 9 (14.5) | 0.085 |
| Previous percutaneous coronary intervention | 20 (10.9) | 11 (9.0) | 9 (14.5) | 0.378 |
| Previous stroke | 22 (12.0) | 14 (11.5) | 8 (12.9) | 0.967 |
| Chronic kidney disease | 2 (1.0) | 1 (0.8) | 1 (1.6) | 1.000 |
| **Biochemical test** | | | | |
| Hb, g/L | 147.97 (13.51) | 146.85 (14.25) | 150.16 (11.73) | 0.117 |
| Plt, 10⁹/L | 226.88 (59.72) | 228.84 (64.03) | 223.00 (50.44) | 0.532 |
| Alb, g/L | 45.99 (3.01) | 45.80 (3.07) | 46.35 (2.88) | 0.238 |
| Cr, µmol/L | 69.52 (47.68) | 65.72 (15.82) | 76.99 (78.99) | 0.13 |
| LDL, mmol/L | 2.33 (0.92) | 2.37 (0.92) | 2.24 (0.93) | 0.375 |
| NT-pro-BNP, pg/ml | 80.87 (127.03) | 82.81 (134.39) | 76.80 (110.94) | 0.768 |
| CRP, mg/L | 1.36 (2.03) | 1.34 (2.15) | 1.40 (1.81) | 0.866 |
| Glucose, mmol/L | 6.82 (2.61) | 6.95 (2.93) | 6.57 (1.83) | 0.354 |
| **TTE** |  |  |  |  |
| LVEF, % | 64.25 (4.54) | 64.23 (4.74) | 64.29 (4.16) | 0.932 |
| **Lesion characteristics** | | | | |
| Target lesion |  |  |  | 0.652 |
| Left anterior descending | 123 (66.8) | 80 (65.6) | 43 (69.4) |  |
| Left circumflex | 27 (14.7) | 20 (16.4) | 7 (11.3) |  |
| Right coronary artery | 34 (18.5) | 22 (18.0) | 12 (19.4) |  |
| Pre TIMI flow grade ≥ 3 | 164 (89.6) | 106 (87.6) | 58 (93.5) | 0.414 |
| Type B2/C | 109 (59.6) | 61 (50.4) | 48 (77.4) | 0.001 |
| Severe angulation | 23 (12.6) | 10 (8.3) | 13 (21.0) | 0.027 |
| Severe calcification | 13 (7.1) | 7 (5.8) | 6 (9.7) | 0.505 |
| Eccentric | 170 (92.9) | 110 (90.9) | 60 (96.8) | 0.324 |
| **ROI characteristics** |  |  |  |  |
| MLD, mm | 1.30 (0.35) | 1.30 (0.34) | 1.30 (0.37) | 0.979 |
| Reference lumen diameter of MLD, mm | 2.99 (0.51) | 2.99 (0.53) | 3.00 (0.48) | 0.89 |
| Diameter stenosis, % | 56.23 (10.26) | 56.12 (9.89) | 56.44 (11.05) | 0.845 |
| Area stenosis, % | 79.79 (10.23) | 79.78 (9.00) | 79.82 (12.37) | 0.978 |
| Lesion length, mm | 28.89 (11.97) | 26.96 (11.76) | 32.67 (11.55) | 0.002 |
| RWS_max_, % | 13.24 (2.69) | 12.42 (1.95) | 14.84 (3.20) | <0.001 |
| **Procedural characteristics** | | | | |
| Transradial intervention, n (%) | 178 (96.7) | 118 (96.7) | 60 (96.8) | 1.000 |
| Lesion pre-treatment before DCB |  |  |  |  |
| Type of predilation balloon |  |  |  | 0.401 |
| Cutting or scoring balloon | 166 (90.2) | 112 (91.8) | 54 (87.1) |  |
| Non-Compliant Balloon | 15 (8.2) | 9 (7.4) | 6 (9.7) |  |
| Semi-Compliant Balloon | 3 (1.6) | 1 (0.8) | 2 (3.2) |  |
| No. of pre-dilation | 2.68 (1.58) | 2.52 (1.32) | 2.98 (1.99) | 0.083 |
| Final pre-dilation balloon diameter, mm | 2.94 (0.35) | 2.94 (0.37) | 2.95 (0.29) | 0.895 |
| Final pre-dilation balloon length, mm | 11.19 (1.85) | 11.29 (1.71) | 11.00 (2.13) | 0.35 |
| Max pre-dilation pressure, atm | 12.50 (3.29) | 12.44 (3.23) | 12.61 (3.44) | 0.756 |
| DCB |  |  |  |  |
| DCB diameter, mm | 30.23 (4.04) | 30.65 (3.39) | 29.40 (5.01) | 0.047 |
| DCB length, mm | 19.81 (6.46) | 20.09 (5.61) | 19.26 (7.90) | 0.411 |
| DCB/artery ratio, % | 110.62 (47.46) | 112.05 (32.70) | 107.81 (68.01) | 0.568 |
| DCB/lesion ratio, % | 147.11 (104.81) | 143.47 (49.98) | 154.27 (167.10) | 0.51 |
| DCB/length ratio, % | 79.71 (37.47) | 86.50 (36.53) | 66.33 (35.95) | <0.001 |
| DCB inflation pressure, atm | 10.88 (3.28) | 10.60 (3.10) | 11.46 (3.58) | 0.094 |
| DCB inflation duration, s | 53.42 (31.94) | 60.53 (28.14) | 38.44 (34.44) | <0.001 |
| Fluoroscopy time, min | 26.12 (14.86) | 23.80 (14.98) | 30.69 (13.61) | 0.003 |
| Contrast medium used, mL | 160.43 (33.71) | 158.47 (36.55) | 164.32 (27.08) | 0.278 |

**Supplemental Table 4: Baseline Characteristics of Patients in Sensitivity Analysis 2**

| **Variable** | **All patients**  **(n=160)** | **Non-Severe Dissection**  **(n=121)** | **Severe Dissection**  **(n=39)** | ***P* value** |
| --- | --- | --- | --- | --- |
| age (y) | 57.17 (9.95) | 56.89 (9.81) | 58.03 (10.47) | 0.538 |
| Male | 116 (72.5) | 85 (70.2) | 31 (79.5) | 0.359 |
| BMI (kg/m2) | 25.81 (3.93) | 25.88 (4.15) | 25.59 (3.18) | 0.694 |
| Cigarette smoking, |  |  |  | 0.104 |
| Current smoker | 45 (28.1) | 29 (24.0) | 16 (41.0) |  |
| Former smoker | 25 (15.6) | 21 (17.4) | 4 (10.3) |  |
| Never smoked | 90 (56.2) | 71 (58.7) | 19 (48.7) |  |
| Clinical presentation |  |  |  | 0.001 |
| Asymptomatic ischemia | 27 (17.0) | 24 (19.8) | 3 (7.9) |  |
| Stable angina | 46 (28.9) | 37 (30.6) | 9 (23.7) |  |
| Unstable angina | 82 (51.6) | 60 (49.6) | 22 (57.9) |  |
| Post myocardial infarction (more than 6 months) | 4 (2.5) | 0 (0.0) | 4 (10.5) |  |
| Diabetes | 46 (28.7) | 35 (28.9) | 11 (28.2) | 1.000 |
| Hypertension | 99 (61.9) | 76 (62.8) | 23 (59.0) | 0.811 |
| Hyperlipidemia | 113 (70.6) | 86 (71.1) | 27 (69.2) | 0.986 |
| Previous myocardial infarction | 13 (8.1) | 7 (5.8) | 6 (15.4) | 0.116 |
| Previous percutaneous coronary intervention | 17 (10.6) | 11 (9.1) | 6 (15.4) | 0.418 |
| Previous stroke | 18 (11.2) | 12 (9.9) | 6 (15.4) | 0.517 |
| Chronic kidney disease | 1 (0.6) | 1 (0.8) | 0 (0.0) | 1.000 |
| **Biochemical test** | | | | |
| Hb, g/L | 147.22 (14.19) | 146.83 (14.68) | 148.41 (12.63) | 0.548 |
| Plt, 10⁹/L | 226.16 (60.66) | 227.60 (64.01) | 221.67 (49.33) | 0.597 |
| Alb, g/L | 45.80 (2.98) | 45.73 (3.06) | 45.99 (2.73) | 0.642 |
| Cr, µmol/L | 69.36 (50.58) | 65.60 (15.86) | 81.03 (98.61) | 0.098 |
| LDL, mmol/L | 2.41 (0.95) | 2.41 (0.94) | 2.41 (0.99) | 0.982 |
| NT-pro-BNP, pg/ml | 81.70 (131.15) | 82.21 (134.94) | 80.01 (119.62) | 0.929 |
| CRP, mg/L | 1.30 (1.95) | 1.31 (2.14) | 1.25 (1.30) | 0.872 |
| Glucose, mmol/L | 6.85 (2.75) | 6.92 (2.94) | 6.63 (2.04) | 0.562 |
| **TTE** |  |  |  |  |
| LVEF, % | 64.13 (4.67) | 64.19 (4.67) | 63.95 (4.73) | 0.78 |
| **Lesion characteristics** | | | | |
| Target lesion |  |  |  | 0.487 |
| Left anterior descending | 105 (65.6) | 77 (63.6) | 28 (71.8) |  |
| Left circumflex | 26 (16.2) | 22 (18.2) | 4 (10.3) |  |
| Right coronary artery | 29 (18.1) | 22 (18.2) | 7 (17.9) |  |
| Pre TIMI flow grade ≥ 3 | 143 (89.4) | 106 (87.6) | 37 (94.9) | 0.423 |
| Type B2/C | 88 (55.0) | 59 (48.8) | 29 (74.4) | 0.009 |
| Severe angulation | 17 (10.6) | 9 (7.4) | 8 (20.5) | 0.045 |
| Severe calcification | 10 (6.2) | 7 (5.8) | 3 (7.7) | 0.962 |
| Eccentric | 148 (92.5) | 110 (90.9) | 38 (97.4) | 0.396 |
| **ROI characteristics** |  |  |  |  |
| MLD, mm | 1.30 (0.32) | 1.30 (0.33) | 1.30 (0.31) | 0.97 |
| Reference lumen diameter of MLD, mm | 3.00 (0.52) | 2.98 (0.53) | 3.05 (0.50) | 0.514 |
| Diameter stenosis, % | 56.15 (9.88) | 55.86 (10.22) | 57.05 (8.84) | 0.512 |
| Area stenosis, % | 79.80 (9.13) | 79.48 (9.44) | 80.79 (8.09) | 0.435 |
| Lesion length, mm | 28.04 (12.16) | 26.76 (11.72) | 32.02 (12.79) | 0.019 |
| RWS_max_, % | 12.91 (2.61) | 12.26 (1.93) | 14.95 (3.33) | <0.001 |
| **Procedural characteristics** | | | | |
| Transradial intervention | 155 (96.9) | 118 (97.5) | 37 (94.9) | 0.766 |
| Lesion pre-treatment before DCB |  |  |  |  |
| Type of predilation balloon |  |  |  | 0.695 |
| Cutting or scoring balloon | 146 (91.2) | 111 (91.7) | 35 (89.7) |  |
| Non-Compliant Balloon | 12 (7.5) | 9 (7.4) | 3 (7.7) |  |
| Semi-Compliant Balloon | 2 (1.2) | 1 (0.8) | 1 (2.6) |  |
| No. of pre-dilation | 2.60 (1.34) | 2.56 (1.31) | 2.74 (1.44) | 0.478 |
| Final pre-dilation balloon diameter, mm | 2.94 (0.36) | 2.93 (0.38) | 2.98 (0.32) | 0.532 |
| Final pre-dilation balloon length, mm | 11.25 (1.80) | 11.27 (1.71) | 11.17 (2.11) | 0.774 |
| Max predilation pressure, atm | 12.52 (3.36) | 12.51 (3.22) | 12.54 (3.80) | 0.959 |
| DCB |  |  |  |  |
| DCB diameter, mm | 30.70 (3.42) | 30.70 (3.36) | 30.71 (3.63) | 0.991 |
| DCB length, mm | 21.19 (5.89) | 20.45 (5.40) | 23.46 (6.80) | 0.005 |
| DCB/artery ratio, % | 111.41 (32.23) | 112.81 (33.00) | 107.05 (29.70) | 0.333 |
| DCB/lesion ratio, % | 142.82 (49.67) | 145.62 (52.25) | 134.15 (39.97) | 0.211 |
| DCB/length ratio, % | 87.23 (35.89) | 88.59 (36.23) | 83.00 (34.95) | 0.399 |
| DCB inflation pressure, atm | 10.59 (3.23) | 10.47 (3.11) | 10.97 (3.58) | 0.4 |
| DCB inflation duration, s | 62.22 (26.42) | 63.42 (25.20) | 58.17 (30.21) | 0.304 |
| Fluoroscopy time, min | 24.43 (14.12) | 22.79 (13.89) | 29.51 (13.77) | 0.009 |
| Contrast medium used, mL | 158.67 (33.25) | 157.59 (35.21) | 162.03 (26.29) | 0.482 |

**Supplemental Table 5: Baseline Characteristics of Patients in Sensitivity Analysis 3**

| **Variable** | **All patients**  **(n=34)** | **Non-Severe Dissection**  **(n=11)** | **Severe Dissection**  **(n=23)** | ***P* value** |
| --- | --- | --- | --- | --- |
| age (y) | 57.22 (7.89) | 59.70 (8.76) | 56.09 (7.41) | 0.237 |
| Male | 24 (70.6) | 6 (54.5) | 18 (78.3) | 0.309 |
| BMI (kg/m2) | 25.48 (3.75) | 25.60 (3.71) | 25.42 (3.85) | 0.899 |
| Cigarette smoking, |  |  |  | 0.591 |
| Current smoker | 10 (29.4) | 3 (27.3) | 7 (30.4) |  |
| Former smoker | 6 (17.6) | 1 (9.1) | 5 (21.7) |  |
| Never smoked | 18 (52.9) | 7 (63.6) | 11 (47.8) |  |
| Clinical presentation |  |  |  | 0.057 |
| Asymptomatic ischemia | 6 (17.6) | 1 (9.1) | 5 (21.7) |  |
| Stable angina | 12 (35.3) | 7 (63.6) | 5 (21.7) |  |
| Unstable angina | 16 (47.1) | 3 (27.3) | 13 (56.5) |  |
| Post myocardial infarction (more than 6 months) | 4 (2.2) | 0 (0.0) | 4 (6.6) |  |
| Diabetes | 10 (29.4) | 2 (18.2) | 8 (34.8) | 0.554 |
| Hypertension | 23 (67.6) | 8 (72.7) | 15 (65.2) | 0.963 |
| Hyperlipidemia | 24 (70.6) | 9 (81.8) | 15 (65.2) | 0.554 |
| Previous myocardial infarction | 3 (8.8) | 0 (0.0) | 3 (13.0) | 0.543 |
| Previous percutaneous coronary intervention | 3 (8.8) | 0 (0.0) | 3 (13.0) | 0.543 |
| Previous stroke | 4 (11.8) | 2 (18.2) | 2 (8.7) | 0.815 |
| Chronic kidney disease | 1 (2.9) | 0 (0.0) | 1 (4.3) | 1.000 |
| **Biochemical test** | | | | |
| Hb, g/L | 151.32 (9.73) | 147.55 (9.42) | 153.13 (9.55) | 0.119 |
| Plt, 10⁹/L | 238.53 (56.58) | 266.27 (55.28) | 225.26 (53.33) | 0.046 |
| Alb, g/L | 45.99 (3.01) | 45.80 (3.07) | 46.35 (2.88) | 0.238 |
| Cr, µmol/L | 67.33 (18.40) | 61.45 (11.19) | 70.14 (20.62) | 0.202 |
| LDL, mmol/L | 2.10 (1.01) | 2.43 (1.37) | 1.92 (0.75) | 0.187 |
| NT-pro-BNP, pg/ml | 82.46 (107.19) | 104.03 (127.67) | 71.16 (96.28) | 0.419 |
| CRP, mg/L | 1.61 (2.18) | 1.43 (1.23) | 1.72 (2.62) | 0.744 |
| Glucose, mmol/L | 6.48 (1.46) | 6.50 (1.57) | 6.47 (1.44) | 0.963 |
| **TTE** |  |  |  |  |
| LVEF, % | 65.12 (4.41) | 65.64 (6.67) | 64.87 (2.96) | 0.642 |
| **Lesion characteristics** | | | | |
| Target lesion |  |  |  | 0.587 |
| Left anterior descending | 24 (70.6) | 9 (81.8) | 15 (65.2) |  |
| Left circumflex | 4 (11.8) | 1 (9.1) | 3 (13.0) |  |
| Right coronary artery | 6 (17.6) | 1 (9.1) | 5 (21.7) |  |
| Pre TIMI flow grade ≥ 3 | 30 (90.9) | 9 (90.0) | 21 (91.3) | 1.000 |
| Type B2/C | 26 (78.8) | 7 (70.0) | 19 (82.6) | 0.726 |
| Severe angulation | 6 (18.2) | 1 (10.0) | 5 (21.7) | 0.755 |
| Severe calcification | 13 (7.1) | 7 (5.8) | 6 (9.7) | 0.505 |
| Eccentric | 31 (93.9) | 9 (90.0) | 22 (95.7) | 1.000 |
| **ROI characteristics** | | | | |
| MLD, mm | 1.32 (0.43) | 1.38 (0.40) | 1.30 (0.46) | 0.61 |
| Reference lumen diameter of MLD, mm | 2.96 (0.45) | 3.07 (0.44) | 2.91 (0.45) | 0.349 |
| Diameter stenosis, % | 55.33 (12.97) | 55.21 (10.58) | 55.40 (14.19) | 0.969 |
| Area stenosis, % | 78.42 (15.21) | 78.92 (9.38) | 78.18 (17.52) | 0.897 |
| Lesion length, mm | 31.36 (10.28) | 26.33 (10.97) | 33.77 (9.23) | 0.047 |
| RWS_max_, % | 14.12 (2.81) | 13.02 (1.99) | 14.64 (3.02) | 0.116 |
| **Procedural characteristics** | | | | |
| Transradial intervention | 33 (97.1) | 10 (90.9) | 23 (100.0) | 0.702 |
| Lesion pre-treatment before DCB |  |  |  |  |
| Type of predilation balloon |  |  |  | 0.727 |
| Cutting or scoring balloon | 29 (85.3) | 10 (90.9) | 19 (82.6) |  |
| Non-Compliant Balloon | 4 (11.8) | 1 ( 9.1) | 3 (13.0) |  |
| Semi-Compliant Balloon | 1 (2.9) | 0 (0.0) | 1 (4.3) |  |
| No. of pre-dilation | 3.03 (2.29) | 2.30 (0.82) | 3.42 (2.71) | 0.216 |
| Final pre-dilation balloon diameter, mm | 2.83 (0.23) | 2.70 (0.20) | 2.89 (0.23) | 0.029 |
| Final pre-dilation balloon length, mm | 10.86 (1.98) | 11.20 (1.55) | 10.68 (2.19) | 0.514 |
| Max pre-dilation pressure, atm | 12.34 (2.57) | 11.60 (2.07) | 12.74 (2.77) | 0.265 |
| Final ballon/artery ratio | 100.61 (89.11) | 82.87 (28.86) | 109.09 (106.30) | 0.431 |
| Final ballon /lesion ratio | 162.00 (224.67) | 106.86 (40.47) | 188.37 (269.68) | 0.33 |
| Final ballon /length ratio | 43.40 (20.93) | 54.55 (30.38) | 38.07 (12.06) | 0.029 |
| Fluoroscopy time, min | 33.56 (15.20) | 35.36 (19.00) | 32.70 (13.41) | 0.639 |
| Contrast medium used, mL | 167.88 (33.43) | 167.27 (43.15) | 168.18 (28.56) | 0.943 |

**Supplemental Table 6**. **Model Performance Comparison With and Without RWSmax**

| **Metric** | **Without RWSmax** | **With RWSmax** | **Improvement** |
| --- | --- | --- | --- |
| **Log-likelihood** | -62.22 | -49.13 | +13.09 |
| **AIC** | 224.43 | 200.27 | -24.16 |
| **BIC** | 381.22 | 360.19 | -21.03 |
| **Hosmer-Lemeshow χ² (p-value)** | 14.24 (p = 0.076) | 9.84 (p = 0.277) | Better calibration |
| **Likelihood ratio test** | - | χ² = 26.17, p < 0.001 | Significant improvement |

**Supplemental Table 7: Predictors of Severe Dissection by Multivariable Regression Analysis in Sensitivity Analysis**

|  | **Sensitive analysis 1** | |  | **Sensitive analysis 2** | |  | **Sensitive analysis 3** | |
| --- | --- | --- | --- | --- | --- | --- | --- | --- |
|  | **Odd Ratio (95% CI)** | ***P* value** |  | **Odd Ratio (95% CI)** | ***P* value** |  | **Odd Ratio (95% CI)** | ***P* value** |
| Type B2/C | **2.34 (1.01 to 5.68)** | **0.050** |  | 2.46 (0.97 to 6.73) | 0.064 |  | 0.60 (0.03 to 6.10) | 0.678 |
| Severe angulation | **3.39 (1.07 to 11.21)** | **0.039** |  | **3.54 (0.99 to 13.03)** | **0.052** |  | 2.79 (0.26 to 71.18) | 0.441 |
| RWS_max_, % | **1.54 (1.31 to 1.90)** | **< 0.001** |  | **1.60 (1.31 to 2.02)** | **< 0.001** |  | ***1.33 (0.95 to 2.04)*** | ***0.131*** |
| DCB/length ratio, % | **0.99 (0.98 to 1.00)** | **0.031** |  | 0.99 (0.98 to 1.00) | 0.212 |  | ***0.95 (0.89 to 1.00)*** | ***0.089*** |
| DCB inflation duration, s | **0.98 (0.97 to 0.99)** | **0.001** |  | **0.98 (0.97 to 1.00)** | **0.069** |  | 0.84 (0.50 to 1.43) | 0.487 |

Sensitivity Analysis 1: Excluding patients with residual stenosis; Sensitivity Analysis 2: Excluding patients who underwent rescue stenting without receiving DCB after lesion pre-treatment; Sensitivity Analysis 3: Patients who underwent rescue stenting without receiving DCB after lesion pre-treatment.
